# Pre-renal or pre-tubular?

**DOI:** 10.12861/jrip.2015.22

**Published:** 2015-11-20

**Authors:** Bijan Roshan

**Affiliations:** Department of Nephrology, Scripps Clinic, 10666 N Torrey Pines Rd, La Jolla, CA, USA

**Keywords:** Acute kidney injury, Renal, Pre-renal, Post-renal

**Implication for health policy/practice/research/medical education:**

Acute kidney injury (AKI) is one of the most common clinical encounters during nephrology inpatient rounds. It is traditionally classified as pre-renal, intrarenal and postrenal categories. However, these terms are often confusing and not very helpful from diagnostic point of view. The frequently used simple and derived urinary indices also give conflicting results among these subtypes. It is suggested to replace these terms with pre-tubular, tubular and post-tubular AKI.

## Introduction

Acute kidney injury (AKI) is one of the most common clinical encounters during nephrology inpatient rounds. Over time, I have seen that, the terms renal, pre-renal, and even post-renal, are causing more confusion than help, when discussing the anatomopathophysiology of AKI to students and even other health care providers.

It is hard to convince a non-nephrologist that the many urinary indices, including fractional excretion of sodium (FeNa) look pre-renal in pure glomerular disease: after all glomeruli are the pillars of main “renal function”, i.e. glomerular filtration rate (GFR). On the other hand, measurements of urinary sodium and creatinine to calculate FeNa are frequently ordered in any AKI when mostly they do not add any to the management. With underlying advanced chronic kidney disease (CKD), FeNa is naturally increased and the validity of the usual indices used to differentiate pre-renal from renal is much reduced. Unfortunately, too much of emphasis is given to FeNa calculation in advanced CKD patients, and frequently no important implication can be made of the calculated value. Frequently in non-oliguric states, FeNa is not needed, as in the absence of diuretics and sodium loosing renal conditions, lack of oliguria per se, would argue more against pre-renal condition than any value that FeNa can imply.

A major cause of confusion is that FeNa is essentially an index of function of renal tubules, and not the GFR, which we define “renal function” with it. With many subtypes of AKI including, pure volume depletion and pure glomerular disease, GFR is decreased, but tubular function of reabsorbing sodium can remain fairly intact in those conditions.

In fact, both simple urinary indices (including urinary sodium, creatinine, osmolality), and derived indices (including excreted fraction of the filtered sodium –FeNa – and renal failure index) that have long been used to differentiate acute tubular necrosis from non-renal conditions (1-5) are meant to determine integrity of “tubular” function.

The terminology of renal, pre-renal, and post-renal is much older than the use of these indices. These indices are chosen to differentiate normal versus abnormal renal tubular handling of sodium. Using the current terminology, it is not surprising that renal arterial as well as glomerular disease (1) without significant involvement of the tubular system have urinary indices compatible with that of pre-renal condition.

A better terminology for pathoanatomophysiologic classification seems to emphasize the tubular versus non-tubular nature of renal failure. Using this terminology, obviously the classical acute tubular injury causing acute renal failure will be considered tubular. Glomerular and renal vascular, as well as conditions associated with low effective intra-arterial volume, will all be properly considered pre-tubular. Early obstructive conditions, inside or outside kidney, without damaging tubular system and tubular handling of sodium, would properly be considered post-tubular in this setting.

Several limitations still apply to even proper use of FeNa. Tubular injury is a common consequence of both pre-tubular and post-tubular conditions and mixed features in the continuum of injury are not uncommon. Also, in chronic renal insufficiency, with reduced GFR and creatinine clearance, to maintain sodium homeostasis, the FeNa is increased.

**Figure 1:**
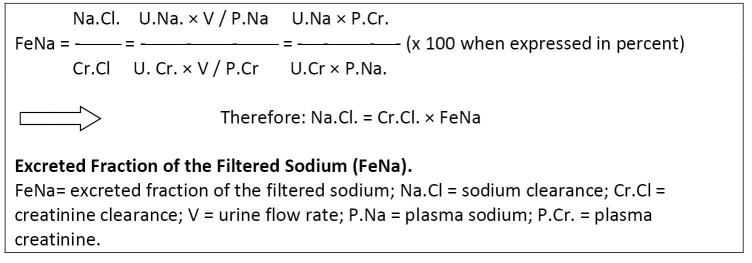
Product of FeNa and creatinine clearance is sodium clearance that is constant under steady sodium homeostasis.

It should remembered that, the classic way to teach FeNa, usually teaches the formula of UNa × PCr/PNa × UCr, without describing where it comes from. To start a mathematical equation, first the understanding of its original non-simplified formula is needed. In this case FeNa implies the fraction of excreted sodium (sodium clearance) per measure of GFR (creatinine clearance). It also has the core implication as to why, for example, with reduced creatinine clearance, FeNa should increase so that sodium clearance stays constant. Thus, calculated FeNa is equal to sodium clearance/creatinine clearance. To maintain the sodium clearance stable under steady states, the product of creatinine clearance and FeNa should stay constant (Figure 1). This means that with decreased but stable creatinine clearance, FeNa should be increased under steady states. Thus, baseline FeNa is usually more than 1.0 in chronic renal insufficiency, which is again indicative of deranged, though compensatory, overall tubular function. Therefore, in most original studies evaluating the validity of FeNa, chronic renal insufficiency patients have been excluded (1-3). The amount of sodium intake also directly affects its clearance and FeNa.

All of these limitations should be considered when using FeNa for evaluation of renal failure.

## Conclusion

Re-naming pre-renal, renal, and even post-renal terms to pre-tubular, tubular, post-tubular, respectively, will better identify the main site of injury/dysfunction in AKI. This change is not mere semantic; it also helps to avoid confusion in transfer of information and correct implication of urinary indices in diagnosis in cases of AKI. The proper cases of AKI, for which, urinary indices are most helpful, include cases of oliguric AKI without underlying advanced CKD.

## Author’s contribution

BR was the single author of the paper.

## Conflict of interests

The author declared no competing interests.

## Ethical considerations

Ethical issues (including plagiarism, data fabrication, double publication) have been completely observed by the author.

## Funding/Support

None.
